# The modified proximal point algorithm in Hadamard spaces

**DOI:** 10.1186/s13660-018-1713-z

**Published:** 2018-05-24

**Authors:** Shih-sen Chang, Lin Wang, Ching-Feng Wen, Jian Qiang Zhang

**Affiliations:** 1 Center for General Education, China Medical University, Taichung, Taiwan; 2 College of Statistics and Mathematics, Yunnan University of Finance and Economics, Kunming, China; 3 Center for Fundamental Science, Kaohsiung Medical University, Kaohsiung, Taiwan

**Keywords:** 47H09, 47J25, Hadamard space, $\operatorname{CAT}(0)$ space, Moreau–Yosida resolvent, Implicit iterative rule, Proximal point algorithm, Variational inequality

## Abstract

The purpose of this paper is to propose a modified proximal point algorithm for solving minimization problems in Hadamard spaces. We then prove that the sequence generated by the algorithm converges strongly (convergence in metric) to a minimizer of convex objective functions. The results extend several results in Hilbert spaces, Hadamard manifolds and non-positive curvature metric spaces.

## Introduction

Let $(X, d)$ be a metric space and $f : X \to(- \infty, \infty]$ be a proper and convex function. One of the most important problems in convex analysis is the convex optimization problem to find $x^{*} \in X$ such that

$$f \bigl(x^{*}\bigr) = \min_{y \in X} f(y). $$

We denote by $\operatorname {argmin}_{y \in X} f (y)$ the set of minimizers of *f* in *X*.

Convex optimization provides us with algorithms for solving a variety of problems which may appear in sciences and engineering. One of the most popular methods for approximation of a minimizer of a convex function is the *proximal point algorithm* (PPA), which was introduced by Martinet [1] and Rockafellar [2] in Hilbert spaces. Indeed, let *f* be a proper, convex and lower semicontinuous function on a real Hilbert space *H* which attains its minimum. The PPA is defined by $x_{1} \in H$ and

$$x_{n+1} =\mathop{\operatorname {argmin}}_{y \in H} \biggl(f(y) + \frac{1}{2\lambda_{n}} \Vert y - x_{n} \Vert ^{2}\biggr),\quad \lambda_{n} > 0, \forall n \ge1. $$

It was proved that the sequence $\{x_{n}\}$ converges weakly to a minimizer of *f* provided $\sum_{n =1}^{\infty}\lambda_{n} = \infty$. However, as shown by Güer [3], the PPA does not necessarily converges strongly (i.e., convergence in metric) in general. For getting the strong convergence of the proximal point algorithm, Xu [4] and Kamimura and Takahashi [5] introduced a Halpern-type regularization of the proximal point algorithm in Hilbert spaces. They proved the strong convergence of Halpern proximal point algorithm under some certain conditions on the parameters.

Recently, many convergence results by PPA for solving optimization problems have been extended from the classical linear spaces such as Euclidean spaces,Hilbert spaces and Banach spaces to the setting of manifolds [6–9]. The minimizers of the objective convex functionals in the spaces with nonlinearity play a crucial role in the branch of analysis and geometry.

In 2013, Bačák [10] introduced the PPA in a $\operatorname{CAT}(0)$ space $(X, d)$ as follows: $x_{1} \in X$ and

$$x_{n+1} = \mathop{\operatorname {argmin}}_{y \in X} \biggl(f(y) + \frac{1}{2\lambda_{n}}d(y, x_{n})^{2}\biggr),\quad \lambda_{n} > 0, \forall n \ge1. $$

Based on the concept of Fejér monotonicity, it was shown that if *f* has a minimizer and $\sum_{n =1}^{\infty}\lambda_{n} = \infty $, then $\{x_{n}\}$ Δ-converges to its minimizer (see also [11]).

In 2015 Cholamjiak [12] presented the modified PPA by Halpern iteration and then prove strong convergence theorem in the framework of $\operatorname{CAT}(0)$ spaces.

Very recently, Khatibzadeh et al. [13] presented a Halpern-type regularization of the proximal point algorithm, under suitable conditions they proved that the sequence generated by the algorithm converges strongly to a minimizer of the convex function in Hadamard spaces.

It is therefore, in this work, to continue along these lines and by using the viscosity implicit rules to introduce the modified PPA in Hadamard space for solving minimization problems. We prove that the sequence generated by the algorithm converges strongly to a minimizer of convex objective functions. The results presented in the paper extend and improve the main results of Martinet [1], Rockafellar [2] Bačák [10], Cholamjiak [12], Xu [4], Kamimura and Takahashi [5], Khatibzadeh et al. [13, Theorem 4.4].

## Preliminaries and lemmas

In order to prove the main results, the following notions, lemmas and conclusions will be needed.

Let $(X; d)$ be a metric space and let $x, y \in X$. A geodesic path joining *x* to *y* is an isometry $c : [0, d(x; y)] \to X$ such that $c(0) = x$, $c(d(x; y)) = y$. The image of a geodesic path joining *x* to *y* is called a geodesic segment between *x* and *y*. The metric space $(X; d)$ is said to be a geodesic space, if every two points of *X* are joined by a geodesic, and *X* is said to be uniquely geodesic space, if there is exactly one geodesic joining *x* and *y* for each $x, y \in X$.

A geodesic space $(X; d)$ is a $\operatorname{CAT}(0)$ space, if and only if

2.1 $$ d^{2}\bigl((1-t)x \oplus t y, z\bigr)\le(1-t) d^{2}(x, z) + t d^{2}(y,z) - t (1-t) d^{2}(x, y) $$

for all $x, y,z \in X$ and all $t \in[0, 1]$ [14].

It is well known that any complete and simply connected Riemannian manifold having non-positive sectional curvature is a $\operatorname{CAT}(0)$ space. Other examples of $\operatorname{CAT}(0)$ spaces include pre-Hilbert spaces [15], *R*-trees, Euclidean buildings [16]. A complete $\operatorname{CAT}(0)$ space is often called a *Hadamard space*. We write $(1-t)x \oplus ty$ for the unique point *z* in the geodesic segment joining from *x* to *y* such that $d(x, z) = td(x, y)$ and $d(y, z) = (1-t)d(x, y)$. We also denote by $[x, y]$ the geodesic segment joining *x* to *y*, that is, $[x, y] = \{(1-t) x \oplus ty: 0 \le t \le 1 \}$. A subset *C* of a $\operatorname{CAT}(0)$ space is convex if $[x, y] \subset C$ for all $x, y \in C$.

For a thorough discussion of $\operatorname{CAT}(0)$ spaces, some fundamental geometric properties and important conclusions, we refer to Bridson and Haefliger [15, 16].

The following lemmas play an important role in proving our main results.

### Lemma 2.1

([17])

*Let* *X* *be a* $\operatorname{CAT}(0)$ *space*. *For all* $x, y, z\in X$ *and* $t, s \in[0,1]$, *we have the following*:

1. $d(t x\oplus(1-t)y, z)\leq t d(x,z) +(1-t) d(y,z)$;
2. $d(t x\oplus(1-t)y, s x\oplus(1-s)y)= |t -s| d(x,y)$;
3. $d(t x\oplus(1- t)y, t u\oplus(1- t)w)\leq t d(x, u) +(1- t) d(y, w)$.

Berg and Nikolaev [18] introduced the following concept of quasi-linearization in $\operatorname{CAT}(0)$ space *X*:

- Denote a pair $(a,b)\in X\times X$ by $\overrightarrow{ab}$ and call it a *vector*. *Quasi-linearization* in $\operatorname{CAT}(0)$ space *X* is defined as a mapping $\langle\cdot,\cdot\rangle: (X\times X)\times(X\times X)\to\mathbb {R}$ such that
  2.2 $$ \langle\overrightarrow{ab},\overrightarrow{cd}\rangle=\frac {1}{2} \bigl(d^{2}(a,d)+d^{2}(b,c)-d^{2}(a,c)-d^{2}(b,d) \bigr) $$
  for all $a,b,c,d\in X$.
- We say that *X* satisfies the Cauchy–Schwarz inequality if
  2.3 $$ \langle\overrightarrow{ab}, \overrightarrow{cd} \rangle\le d(a, b) d(c, d),\quad \forall a, b, c, d \in X. $$
  It is well known [18, Corollary 3] that a geodesically connected metric space is a $\operatorname{CAT}(0)$ space if and only if it satisfies the Cauchy–Schwarz inequality.
- By using quasi-linearization, Ahmadi Kakavandi [19] proved that $\{x_{n}\}$ Δ-converges to $x \in X$ if and only if
  2.4 $$ \limsup_{n \to\infty}\langle\overrightarrow{xx_{n}}, \overrightarrow {xy}\rangle\le0,\quad \forall y \in X. $$
- Let *C* be a nonempty closed convex subset of a complete $\operatorname{CAT}(0)$ space *X* (i.e., a Hadamard space). The *metric projection* $P_{C}: X\to C$ is defined by
  2.5 $$ u=P_{C}(x) \quad \Longleftrightarrow \quad d(u,x)=\inf\bigl\{ d(y,x):y \in C\bigr\} ,\quad x\in X. $$

### Lemma 2.2

([18])

*Let* *C* *be a nonempty closed and convex subset of a Hadamard space* *X*, $x\in X$ *and* $u\in C$. *Then* $u=P_{C}(x)$ *if and only if*

2.6 $$ \langle\overrightarrow{yu},\overrightarrow{ux}\rangle\geq0,\quad \forall y\in C. $$

Let *C* be a convex subset of a $\operatorname{CAT}(0)$ space *X*. Recall that a function $f : C \to(-\infty, \infty]$ is said to be convex if, for any geodesic $\gamma: [a, b] \to C$, the function $f\circ\gamma$ is convex, i.e.,

2.7 $$ f\bigl(\gamma[a, b]\bigr): = f\bigl((1-t)a \oplus t b\bigr) \le(1-t) f(a) + t f(b), \quad \forall a, b \in C\mbox{ and }t \in(0, 1). $$

Some important examples of convex functions can be found in [15]. For $r > 0$, define the Moreau–Yosida resolvent of *f* in $\operatorname{CAT}(0)$ spaces as

2.8 $$ J_{r} (x) = \mathop{\operatorname {argmin}}_{y \in X}\biggl(f(y) + \frac{1}{2r} d^{2}(y,x)\biggr) $$

for all $x \in X$ (see [20]). The mapping $J_{r}$ is well defined for all $r > 0$ (see [20]).

### Lemma 2.3

([11])

*Let* $(X, d)$ *be a Hadamard space and* $f : X \to (-\infty, \infty]$ *be a proper*, *convex and lower semicontinuous function*. *Then*, *for every* $r > 0$:

1. *the resolvent* $J_{r}$ *is firmly nonexpansive*, *that is*,
  $$d(J_{r} x, J_{r} y) \le d\bigl((1 - \lambda)x \oplus \lambda J_{r} x, (1 - \lambda )y \oplus\lambda J_{r} y\bigr) $$
  *for all* $x, y \in X$ *and for all* $\lambda\in(0, 1)$;
2. *the set* $\operatorname{Fix}(J_{r})$ *of fixed points of the resolvent* $J_{r}$ *associated with* *f* *coincides with the set* $\operatorname {argmin}_{y\in X} f(y)$ *of minimizers of* *f*.

### Remark 2.4

Every firmly nonexpansive mapping is nonexpansive. Hence $J_{r}$ is a nonexpansive mapping.

### Lemma 2.5

([21])

*Let* *X* *be a* $\operatorname{CAT}(0)$ *space*, *C* *be a nonempty closed and convex subset of* *X* *and* $T:C\rightarrow C$ *be a nonexpansive mapping*. *For any contraction* $\phi:C\rightarrow C$ *and* $t \in(0,1)$, *let* $x_{t} \in C$ *be the unique fixed point of the contraction* $x \mapsto t f (x)\oplus(1-t)Tx$, *i*.*e*.,

2.9 $$ x_{t} = t \phi(x_{t} )\oplus(1 -t)Tx_{t}. $$

*Then* $\{x_{t}\}$ *converge strongly as* $t \to0$ *to a point* $x^{*}$ *such that*

$$x^{*} = P_{\operatorname{Fix}(T)} \phi\bigl(x^{*}\bigr), $$

*which is the unique solution to the following variational inequality*:

2.10 $$ \bigl\langle \overrightarrow{x^{*}\phi\bigl(x^{*}\bigr)}, \overrightarrow{xx^{*}}\bigr\rangle \ge 0,\quad \forall x \in \operatorname{Fix}(T). $$

### Lemma 2.6

([22])

*Let* $\{a_{n}\}$ *be a sequence of nonnegative real numbers satisfying*

2.11 $$ a_{n+1} \leq(1-\gamma_{n})a_{n} + \delta_{n} $$

*for all* $n \geq0$, *where* $\{\gamma_{n}\}$ *is a sequence in* $(0,1)$ *and* $\{\delta_{n}\}$ *is a sequence in* $\mathbb{R}$ *such that*:

- $\sum_{n=1}^{\infty}\gamma_{n} = \infty$;
- $\limsup_{n \to\infty} \frac{\delta_{n}}{\gamma_{n}}\leq0$ *or* $\sum_{n=1}^{\infty}|\delta_{n}| < \infty$.

*Then* $\lim_{n \to\infty} a_{n}=0$.

## The main results

Now, we are in a position to give the main results in this paper.

### Theorem 3.1

*Let* *C* *be a nonempty closed and convex subset of a Hadamard space* *X*. *Let* $r > 0$, *and* $f: C \to(-\infty, \infty]$ *be a proper*, *convex and lower semicontinuous function with* $\operatorname{Fix}(J_{r}) \neq \emptyset$, *where* $J_{r}$ *is the Moreau–Yosida resolvent of* *f* *defined by*

$$J_{r}(x)= \mathop{\operatorname {argmin}}_{y \in C}\biggl(f(y) + \frac{1}{2r} d^{2}(y,x)\biggr). $$

*Let* $\phi: C\rightarrow C$ *be a contraction with the contractive coefficient* $k\in[0,1)$ *and*, *for arbitrary initial point* $x_{0}\in C$, *let* $\{x_{n}\}$ *be the implicit iterative sequence generated by*

3.1 $$ x_{n+1} =\alpha_{n} \phi(x_{n})\oplus(1- \alpha_{n}) J_{r}\bigl(\beta_{n} x_{n} \oplus(1 - \beta_{n}) x_{n+1}\bigr) $$

*for all* $n\geq0$, *where* $\alpha_{n} \in(0, 1)$, $\beta_{n} \in[0,1]$ *satisfy the following conditions*:

- $\lim_{n\to\infty}\alpha_{n}=0$;
- $\sum_{n=0}^{\infty} \alpha_{n}=\infty$;
- $\frac{|\alpha_{n}-\alpha_{n-1}|}{\alpha_{n}^{2}} \to0$ *as* $n \to\infty$.

*Then the sequence* $\{x_{n}\}$ *converges strongly to* $x^{*}=P_{\operatorname{Fix}(J_{r})} \phi (x^{*})$, *which is a fixed point of* $J_{r}$ (*therefore*, *by Lemma *2.3, *it is a minimizer of* *f*) *and it is also a solution of the following variational inequality*:

$$\bigl\langle \overrightarrow{x^{*}\phi\bigl(x^{*}\bigr)},\overrightarrow{x x^{*}} \bigr\rangle \geq0, \quad \forall x\in \operatorname{Fix}(J_{r}). $$

### Proof

We divide the proof into four steps.

*Step* 1. First, we prove that the sequence $\{x_{n}\}$ defined by (3.1) is well defined. In fact, for arbitrarily given $u \in C$, the mapping

3.2 $$ x \mapsto T_{u} (x): = \alpha\phi(u) \oplus(1-\alpha)J_{r} \bigl(\beta u \oplus(1 - \beta) x\bigr), \quad x \in C, \mbox{and }\alpha\in(0, 1), \beta\in[0, 1] $$

is a contraction with the contractive constant $1- \alpha$.

Indeed, it follows from Lemma 2.1 and Lemma 2.3 that, for any $x, y \in C$,

$$\begin{aligned}& d(T_{u} x, T_{u} y) \\& \quad = d\bigl(\alpha\phi(u) \oplus(1-\alpha)J_{r}\bigl(\beta u \oplus(1 - \beta) x\bigr), \alpha\phi(u) \oplus(1-\alpha)J_{r}\bigl( \beta u \oplus(1 - \beta) y\bigr)\bigr) \\& \quad \le (1- \alpha) d\bigl(J_{r}\bigl(\beta u \oplus(1 - \beta) x \bigr), J_{r}\bigl(\beta u \oplus(1 - \beta) y\bigr)\bigr) \\& \quad \le (1- \alpha) (1 - \beta) d(x,y) \le (1- \alpha) d(x,y). \end{aligned}$$

This implies that the mapping $T_{u}: C \to C $ is a contraction. Hence the implicit iterative sequence $\{x_{n}\}$ defined by (3.1) is well defined.

*Step* 2. Next, we prove that $\{x_{n}\}$ is bounded.

In fact, taking $p\in \operatorname{Fix}(J_{r})$, we have

$$\begin{aligned}& d(x_{n+1},p) \\& \quad = d\bigl(\alpha_{n} \phi(x_{n})\oplus(1- \alpha_{n}) J_{r}\bigl(\beta_{n} x_{n} \oplus(1 - \beta_{n}) x_{n+1}\bigr), p\bigr) \\& \quad \leq \alpha_{n} d\bigl(\phi(x_{n}),p\bigr)+(1- \alpha_{n})d\bigl(J_{r}\bigl(\beta_{n} x_{n}\oplus(1 - \beta_{n}) x_{n+1}\bigr), p\bigr) \\& \quad \leq \alpha_{n} \bigl(d\bigl(\phi(x_{n}),\phi(p) \bigr)+d\bigl(\phi(p),p\bigr)\bigr)+(1-\alpha _{n})d \bigl(J_{r}\bigl(\beta_{n} x_{n}\oplus(1 - \beta_{n}) x_{n+1}\bigr), J_{r}(p)\bigr) \\& \quad \leq \alpha_{n} k d(x_{n},p)+\alpha_{n} d \bigl(\phi(p),p\bigr)+(1-\alpha_{n}) \bigl(\beta_{n} d(x_{n}, p) + (1 - \beta_{n})d(x_{n+1}, p)\bigr), \end{aligned}$$

which implies that

$$\begin{aligned} d(x_{n+1},p) \le& \frac{1}{\alpha_{n} + (1-\alpha_{n})\beta_{n}}\bigl\{ \bigl(\alpha_{n} k + (1-\alpha_{n})\beta_{n}\bigr) x(x_{n}, p) + \alpha_{n} d\bigl(\phi(p), p\bigr)\bigr\} \\ =& \biggl(1 - \frac{\alpha_{n} (1-k)}{\alpha_{n} + (1-\alpha_{n})\beta_{n}} \biggr)d(x_{n}, p) + \frac{\alpha_{n} (1-k) d(\phi(p), p)}{(\alpha_{n} + (1-\alpha _{n})\beta_{n})(1-k)} \\ \le& \max \biggl\{ d(x_{n}, p), \frac{d(\phi(p), p)}{1-k} \biggr\} . \end{aligned}$$

By induction, we can prove that

$$d(x_{n},p) \le\max \biggl\{ d(x_{0}, p), \frac{d(\phi(p), p)}{1-k} \biggr\} $$

for all $n \ge0$. This implies that $\{x_{n}\}$ is bounded and so are $\{ \phi(x_{n})\}$ and $\{J_{r}(\beta_{n} x_{n}\oplus(1 - \beta_{n}) x_{n+1})\}$.

*Step* 3. Next, we prove that the sequence $\{x_{n}\}$ converges strongly to some point in $\operatorname{Fix}(J_{r})$.

Letting

3.3 $$ z_{n} = \alpha_{n} \phi(z_{n})\oplus(1- \alpha_{n}) J_{r} z_{n} $$

for all $n \ge0$. By Lemma 2.5, the sequence $\{z_{n}\}$ converges strongly as $n \to\infty$ to a point $x^{*} = P_{\operatorname{Fix}(J_{r})} \phi(x^{*})$, which is the unique solution to the following variational inequality:

3.4 $$ \bigl\langle \overrightarrow{x^{*}\phi\bigl(x^{*}\bigr)}, \overrightarrow{xx^{*}}\bigr\rangle \ge 0,\quad \forall x \in \operatorname{Fix}(J_{r}). $$

On the other hand, it follows from (3.1), Lemma 2.3 and Lemma 2.1 that

$$\begin{aligned}& d(x_{n+1}, z_{n}) \\& \quad = d\bigl(\alpha_{n} \phi(x_{n})\oplus(1- \alpha_{n}) J_{r}\bigl(\beta_{n} x_{n} \oplus(1 - \beta_{n}) x_{n+1}\bigr),\alpha_{n} \phi(z_{n})\oplus(1-\alpha_{n}) J_{r} z_{n}\bigr) \\& \quad \le \alpha_{n} d\bigl(\phi(x_{n}), \phi(z_{n})\bigr) + (1 - \alpha_{n})d\bigl(J_{r} \bigl(\beta_{n} x_{n}\oplus(1 - \beta_{n}) x_{n+1}\bigr), J_{r} z_{n}\bigr) \\& \quad \le \alpha_{n} k d(x_{n}, z_{n}) + (1 - \alpha_{n}) \bigl(\beta_{n} d(x_{n}, z_{n}) + (1 - \beta_{n}) d(x_{n+1}, z_{n})\bigr), \end{aligned}$$

which implies that

3.5 $$\begin{aligned} d(x_{n+1}, z_{n}) \le& \frac{\alpha_{n} k + (1-\alpha_{n})\beta_{n} }{\alpha _{n} + (1-\alpha_{n})\beta_{n}} d(x_{n}, z_{n}) \\ =& \biggl(1 - \frac{\alpha_{n} (1-k)}{\alpha_{n} + (1-\alpha_{n})\beta_{n}} \biggr)d(x_{n}, z_{n}) \\ \le& \bigl( 1 - \alpha_{n} (1-k)\bigr) \bigl(d(x_{n}, z_{n-1}) + d( z_{n-1}, z_{n})\bigr) \\ \le& \bigl( 1 - \alpha_{n} (1-k)\bigr) d(x_{n}, z_{n-1}) + d( z_{n-1}, z_{n}). \end{aligned}$$

In order to use Lemma 2.6, it should be proved that

3.6 $$ \limsup_{n \to\infty}\frac{d( z_{n-1}, z_{n})}{\alpha_{n} (1-k)} \le0. $$

In fact, by Lemma 2.1 and Lemma 2.3, we have

$$\begin{aligned}& d(z_{n}, z_{n-1}) \\& \quad = d\bigl(\alpha_{n} \phi(z_{n})\oplus(1- \alpha_{n}) J_{r}z_{n}, \alpha_{n-1} \phi (z_{n-1}) \oplus(1-\alpha_{n-1}) J_{r} z_{n-1}\bigr) \\& \quad \le d\bigl(\alpha_{n} \phi(z_{n})\oplus(1- \alpha_{n}) J_{r}z_{n}, \alpha_{n} \phi (z_{n}) \oplus(1-\alpha_{n}) J_{r} z_{n-1}\bigr) \\& \qquad {} + d\bigl(\alpha_{n} \phi(z_{n})\oplus(1- \alpha_{n}) J_{r}z_{n-1}, \alpha_{n} \phi (z_{n-1})\oplus(1-\alpha_{n}) J_{r}z_{n-1} \bigr) \\& \qquad {} + d\bigl(\alpha_{n} \phi(z_{n-1})\oplus(1- \alpha_{n}) J_{r}z_{n-1}, \alpha _{n-1} \phi(z_{n-1}) \oplus(1-\alpha_{n-1}) J_{r} z_{n-1}\bigr) \\& \quad \le (1-\alpha_{n})d( J_{r}z_{n}, J_{r}z_{n-1}) + \alpha_{n} d\bigl( \phi(z_{n}), \phi (z_{n-1})\bigr) + |\alpha_{n} - \alpha_{n-1}| d\bigl(\phi(z_{n-1}),J_{r} z_{n-1}\bigr) \\& \quad \le (1-\alpha_{n})d( z_{n}, z_{n-1}) + \alpha_{n} k d( z_{n}, z_{n-1}) + | \alpha_{n} - \alpha_{n-1}| M, \end{aligned}$$

where $M = \sup_{n \ge1}d(\phi(z_{n-1}), J_{r} z_{n-1})$, which implies that

$$d(z_{n}, z_{n-1}) \le\frac{1}{\alpha_{n} (1-k)}| \alpha_{n} - \alpha_{n-1}| M. $$

By the condition (c), we have

$$\limsup_{n \to\infty}\frac{d( z_{n-1}, z_{n})}{\alpha_{n} (1-k)} \le \limsup _{n \to\infty}\frac{|\alpha_{n} - \alpha_{n-1}| M}{\alpha_{n}^{2} (1- k)^{2}} =0. $$

Hence (3.6) is proved. By Lemma 2.6 and (3.5), it follows that

$$\lim_{n \to\infty} d(x_{n+1}, z_{n}) = 0. $$

Since $z_{n} \to x^{*} = P_{\operatorname{Fix}(J_{r})}\phi(x^{*})$, this implies that $x_{n} \to x^{*} \in \operatorname{Fix}(J_{r})$. By Lemma 2.3, $x^{*} \in \operatorname {argmin}_{y\in C} f(y)$ and $x^{*}$ is also the unique solution of the variational inequality (3.4).

This completes the proof. □

### Remark

An simple example of a sequence $\{\alpha_{n}\}$ satisfying conditions (a)–(c) is given by $\{\alpha_{n} = 1/n^{\sigma}\}$, where $0 < \sigma< 1$.

Looking at the proof of Theorem 3.1, we only use the fact that the resolvent operator $J_{r}$ is nonexpansive. If we replace the resolvent operator $J_{r}$ with a nonexpansive mapping $T : C \to C$ in Theorem 3.1, then we can obtain the following.

### Theorem 3.2

*Let* *C* *be a nonempty closed and convex subset of a Hadamard space* *X*. *Let* $T:C \to C$ *be a nonexpansive mapping with* $\operatorname{Fix}(T) \neq \emptyset$. *Let* $\phi: C\rightarrow C$ *be a contraction with the contractive coefficient* $k\in[0,1)$ *and*, *for arbitrary initial point* $x_{0}\in C$, *let* $\{x_{n}\}$ *be the implicit iterative sequence generated by*

3.7 $$ x_{n+1} =\alpha_{n} \phi(x_{n})\oplus(1- \alpha_{n}) T\bigl(\beta_{n} x_{n}\oplus(1 - \beta_{n}) x_{n+1}\bigr) $$

*for all* $n\geq0$, *where* $\alpha_{n} \in(0, 1), \beta_{n} \in[0,1]$ *satisfy the following conditions*:

- $\lim_{n\to\infty}\alpha_{n}=0$;
- $\sum_{n=0}^{\infty} \alpha_{n}=\infty$;
- $\frac{|\alpha_{n}-\alpha_{n-1}|}{\alpha_{n}^{2}} \to0$ *as* $n \to\infty$.

*Then the sequence* $\{x_{n}\}$ *converges strongly to* $x^{*}=P_{\operatorname{Fix}(T)} \phi (x^{*})$, *which is a fixed point of* *T* *and it is also a solution of the following variational inequality*:

$$\bigl\langle \overrightarrow{x^{*}\phi\bigl(x^{*}\bigr)},\overrightarrow{x x^{*}} \bigr\rangle \geq0,\quad \forall x\in \operatorname{Fix}(T). $$

Since every Hilbert space is a Hadamard space, the following result can be obtained from Theorem 3.1 immediately.

### Theorem 3.3

*Let* *C* *be a nonempty closed and convex subset of a real Hilbert* *H*. *Let* $r > 0$, *and* $f: C \to(-\infty, \infty]$ *be a proper*, *convex and lower semicontinuous function with* $\operatorname{Fix}(J_{r}) \neq \emptyset$, *where* $J_{r}$ *is the Moreau–Yosida resolvent of* *f* *defined by*

$$J_{r}(x)= \mathop{\operatorname {argmin}}_{y \in C}\biggl(f(y) + \frac{1}{2r} d^{2}(y,x)\biggr). $$

*Let* $\phi: C\rightarrow C$ *be a contraction with the contractive coefficient* $k\in[0,1)$ *and*, *for arbitrary initial point* $x_{0}\in C$, *let* $\{x_{n}\}$ *be the sequence generated by*

3.8 $$ x_{n+1} =\alpha_{n} \phi(x_{n}) + (1- \alpha_{n}) J_{r}\bigl(\beta_{n} x_{n} + (1 - \beta _{n}) x_{n+1}\bigr) $$

*for all* $n\geq0$, *where* $\alpha_{n} \in(0, 1)$, $\beta_{n} \in[0,1]$ *satisfy the conditions* (a)*–*(c) *in Theorem *3.1. *Then the conclusions in Theorem *3.1 *still hold*.

## Applications

In this section, we shall utilize the results presented in the paper to study a class of inclusion problems in Hilbert space.

Let *H* be a real Hilbert space and $f : H \to(- \infty, \infty]$ be a proper and convex lower semicontinuous function. Now we consider the following inclusion problem: to find a point $x^{*} \in H$ such that

4.1 $$ x^{*} \in(\partial f)^{-1}(0), $$

where *∂f* is the subdifferential of *f*. By Fermat’s theorem (see Rockafellar [2]), it is easy to see that

4.2 $$ x^{*} \in(\partial f)^{-1}(0)\quad \Leftrightarrow\quad x^{*} \in \operatorname{Fix}\bigl(J_{r}^{\partial f}\bigr)\quad \Leftrightarrow \quad f\bigl(x^{*}\bigr) = \min_{y \in H} f(y), $$

where $J_{r}^{\partial f}$ is the resolvent associated with *∂f* defined by

4.3 $$ J_{r}^{\partial f}(x) := (I + r \partial f)^{-1}(x),\quad x \in H, r > 0, $$

where *I* stands for the identity mapping on *H*.

We note that, for all $r > 0$, the resolvent mapping $J_{r}^{\partial f}$ is a single-valued nonexpansive mapping.

Therefore the following result can be obtained from Theorem 3.2 immediately.

### Theorem 4.1

*Let* *H* *be a real Hilbert space*, $r > 0$ *and* $f: H \to(-\infty, \infty]$ *be a proper*, *convex and lower semicontinuous function with* $\operatorname{Fix}(J_{r}^{\partial f}) \neq \emptyset$. *Let* $\phi: H\rightarrow H$ *be a contraction with the contractive coefficient* $k\in[0,1)$. *For arbitrary initial point* $x_{0}\in H$, *let* $\{x_{n}\}$ *be the sequence generated by*

4.4 $$ x_{n+1} =\alpha_{n} \phi(x_{n}) + (1- \alpha_{n}) J_{r}^{\partial f}\bigl(\beta_{n} x_{n} + (1 - \beta_{n}) x_{n+1}\bigr),\quad n \ge0, $$

*where* $\alpha_{n} \in(0, 1)$, $\beta_{n} \in[0,1]$ *satisfy the conditions* (a)*–*(c) *in Theorem *3.2. *Then* $\{x_{n}\}$ *converges strongly to a point* $x^{*} \in H$ *which is a solution of inclusion problem* (4.1), *also it is a minimizer of* *f* *in* *H*.

Similarly, by using the same method mentioned above, we can study the *monotone variational inclusion problem* (in short, (MVIP)) in real Hilbert space *H* to find a point $x^{*} \in H$ such that

4.5 $$ 0 \in M\bigl(x^{*}\bigr), $$

where $M: H \to2^{H}$ is a maximal monotone mapping.

It is easy to see that the problem (MVIP) (4.5) is equivalent to find $x^{*} \in H$ such that

$$x^{*} \in \operatorname{Fix}\bigl(J_{r}^{M}\bigr),\quad r > 0, $$

where $J_{r}^{M}$ is the resolvent associated with *M* defined by

4.6 $$ J_{r}^{M} (x) = (I + r M)^{-1}(x),\quad x \in H, $$

which is nonexpansive. Replacing the resolvent $J_{r}^{\partial f}$ by the resolvent $J_{r}^{M}$ in Theorem 4.1, we have the following.

### Theorem 4.2

*Let* *H* *be a real Hilbert* *H*. *Let* $r > 0$, *and* $M: H \to2^{H}$ *be a maximal monotone mapping with* $\operatorname{Fix}(J_{r}^{M}) \neq \emptyset$. *Let* $\phi: H \rightarrow H$ *be a contraction with the contractive coefficient* $k\in[0,1)$. *For arbitrary initial point* $x_{0}\in H$, *let* $\{x_{n}\}$ *be the sequence generated by*

4.7 $$ x_{n+1} =\alpha_{n} \phi(x_{n}) + (1- \alpha_{n}) J_{r}^{M}\bigl(\beta_{n} x_{n} + (1 - \beta_{n}) x_{n+1}\bigr),\quad n \ge0, $$

*where* $\alpha_{n} \in(0, 1)$, $\beta_{n} \in[0,1]$ *satisfy the conditions* (a)*–*(c) *in Theorem *4.1. *Then* $\{x_{n}\}$ *converges strongly to a point* $x^{*} \in H$ *which is a solution of inclusion problem* (4.5).
